# Nearly Fatal Torsade de Pointes with Sotalol

**Published:** 2003-10-01

**Authors:** Bernhard Strohmer, Christiana Schernthaner, Maximilian Pichler

**Affiliations:** Department of Cardiology, Landeskliniken Salzburg, Austria

A 75-year-old woman with paroxysmal atrial fibrillation (AF) experienced recurrent seizures at home. Holter monitoring (Figure 1) (each line 50 sec) showed repetitive runs of torsade de pointes (TdP), degenerating into ventricular fibrillation and ventricular tachycardia (VT). Abrupt asytole heralded end of electrical activity and life. Amazingly, 6 min after cardiac arrest a slow ventricular escape rhythm arose spontaneously without resuscitation. At baseline, repolarisation was markedly prolonged (QTc>660 msec) and ventricular bigeminy triggered short bursts of TdP after "long-short" sequences. No hypokalemia or renal dysfunction was present. Following intensive treatment (sedation, magnesium iv, acceleration of heart rate) the patient recovered without neurological deficit. Except left ventricular hypertrophy and incomplete left bundle branch block the results of angiography, electrophysiological study and ajmaline test were normal. There was no family history of sudden death. Months ago a cardioversion attempt with ibutilide triggered polymorphic VT. Therapy with metoprolol (95 mg/day) was discontinued due to poor efficacy of rhythm control. Thus, sotalol (240 mg/day) was initiated in-hospital without signs of QT prolongation within 4 days (QT 416, QTc 432 msec). However, two weeks later the patient presented with an "idiosyncratic" proarrhythmic response to sotalol (I_Kr_-blocking drug) and a life-threatening arrhythmia [1]. There is growing evidence that drug-induced long QT syndrome (LQTS) may be due to "silent" mutations on LQT genes [2,3]. Although not proven by molecular analysis, our case seems to resemble a subclinical, inherited form of LQTS that makes the patient vulnerable to the QT-prolonging effects of a variety of cardiac and noncardiac drugs. The concept of "repolarization reserve" suggests that any factor that impairs the repolarizing currents renders TdP very likely when I_Kr_-blocking drugs are used. Avoiding torsadogenic drugs should basically prevent recurrence of TdP. However, an implantable cardioverter-defibrillator was placed for safety reasons [4]. During a follow-up of more than two years a few non-sustained episodes occurred, the longest, a short-coupled polymorphic VT lasted for 25 beats resulting in a diverted shock.

## Figures and Tables

**Figure 1:**
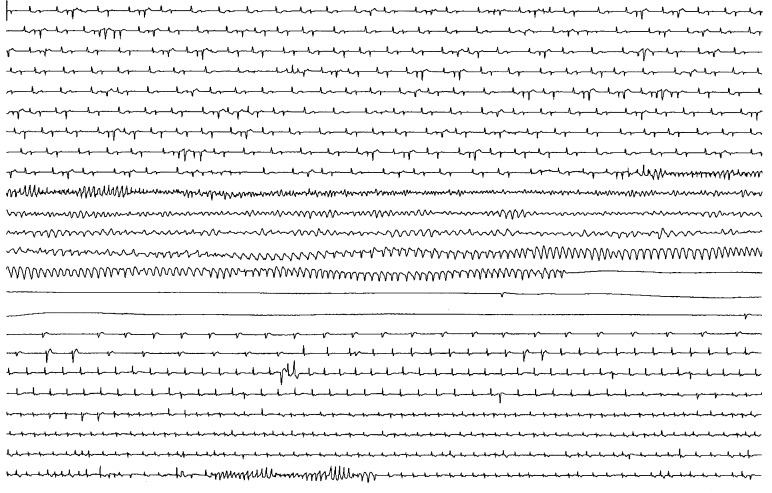
Holter tracing (each line 50 sec)
